# Esthetic Rehabilitation Using Magnet-Retained Cheek Plumper Prosthesis

**DOI:** 10.1155/2020/2769873

**Published:** 2020-08-26

**Authors:** Alisha Rewari, Nupur Dabas, Reshu Sanan, Shefali Phogat, Sumit Singh Phukela, Monika Vigarniya

**Affiliations:** ^1^Department of Prosthodontics, SGT University, India; ^2^Department of Prosthodontics, Faculty of Dental Sciences, SGT University, India; ^3^Shaheed Hasan Khan Mewati Government Medical College, India

## Abstract

*Summary*. Prosthetic rehabilitation of a completely edentulous patient is no more confined to replacement of missing teeth. Long span of edentulism and ageing leads to loss of support of the facial musculature, which is of great concern in treating completely edentulous patients. Flaccid facial musculature eventually leads to sunken cheeks and unesthetic appearance, causing a negative impact on psychological well-being of the patient. The use of conventional complete dentures can restore the loss to some extent, but in some cases, additional support is required. The present clinical report exemplifies the use of magnet-retained detachable maxillary cheek plumper prosthesis in a completely edentulous patient with sunken cheeks.

## 1. Introduction

Ageing has an impact on facial esthetics due to loss of alveolar process and teeth, loss of muscle tonicity, loss of elasticity of the skin, and impairment of function [1]. Prosthetic rehabilitation of a completely edentulous patient is no more confined to replacement of missing teeth. Any and every prosthesis should replace both function and esthetics. Conventional complete dentures with appropriate flange extensions customarily support the overlying lips and cheeks.

But, certain factors like early loss of posterior teeth, thinning of the tissues, and weight loss may cause concavities below the malar bone or slumped cheeks, which affects esthetics making the patient look much older than their age [2]. This also has a negative impact on their social and professional lives leading to a detrimental psychological effect on the patient. Thus, to support the sunken cheeks, a prosthesis known as cheek plumper can be used. A conventional cheek plumper, which is a single unit prosthesis that adds on to the weight of the denture, increases the mesiodistal width of the prosthesis, thereby making its insertion difficult in patients with limited mouth opening [3]. Moreover, its long-term use can cause muscle fatigue. Thus, to combat this situation, a detachable cheek plumper can be fabricated.

The present clinical report exemplifies the use of magnets to support a detachable cheek plumper prosthesis in a completely edentulous patient with sunken cheeks.

## 2. Case Report

**Figure 1:**
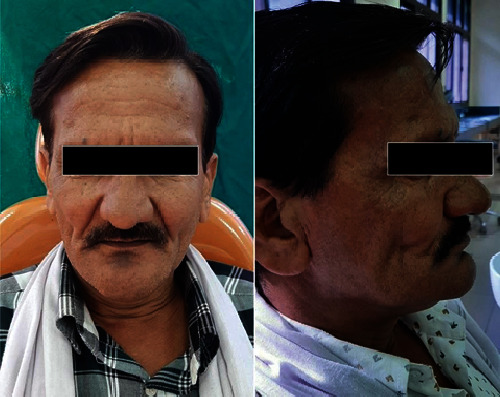
Straight profile and side profile of the edentulous patient showing sunken cheeks.

A 42-year-old male patient reported to the Department of Prosthodontics, Faculty of Dental Sciences, SGT University, with the chief complaint of missing teeth and poor esthetics since 3 years. He lost some of his teeth due to a road accident while some due to poor periodontal health. On intraoral examination, the patient had completely edentulous, low, well-rounded maxillary and mandibular arch. Extraoral examination revealed wrinkling of the skin and unsupported musculature leading to sunken cheeks. The patient was very conscious of his appearance and desired a prosthesis which would make his face look fuller and healthier. Keeping the patient's demand in mind, fabrication of maxillary and mandibular complete dentures with intraoral magnet-retained, detachable cheek plumpers attached to the maxillary denture was planned (Figure 1). The steps of fabrication were as follows.

**Figure 2:**
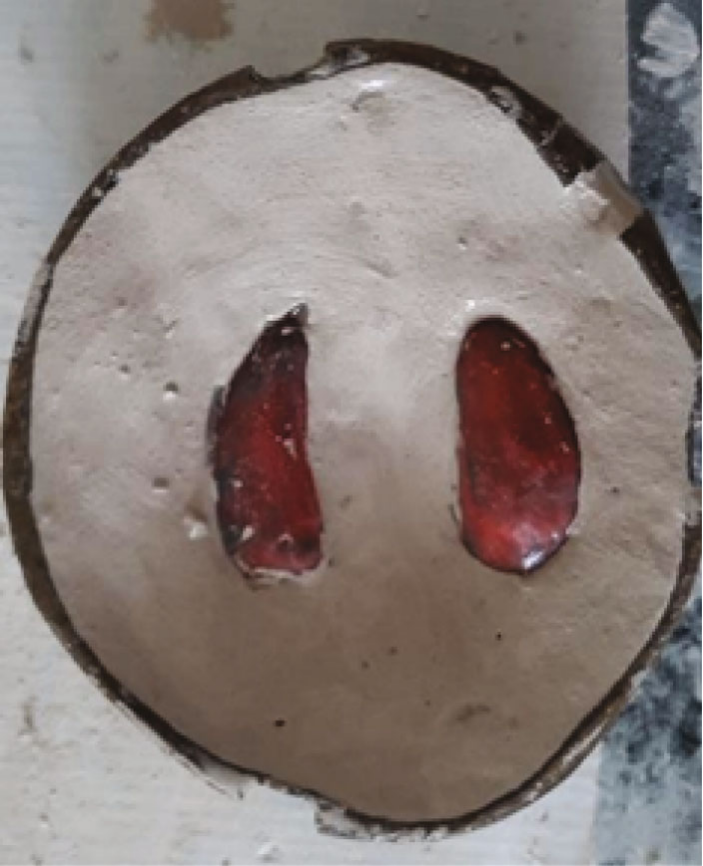
Investment of cheek plumper (wax pattern).

**Figure 3:**
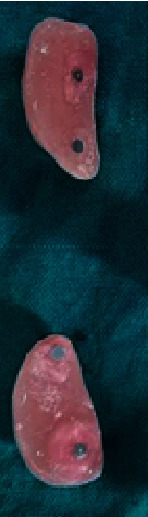
Cheek plumpers showing magnetic attachments.

**Figure 4:**
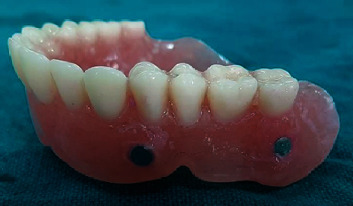
Buccal flange of maxillary denture showing magnetic attachments.

**Figure 5:**
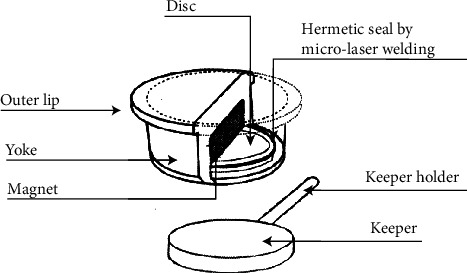
Magfit™ DX600.

**Figure 6:**
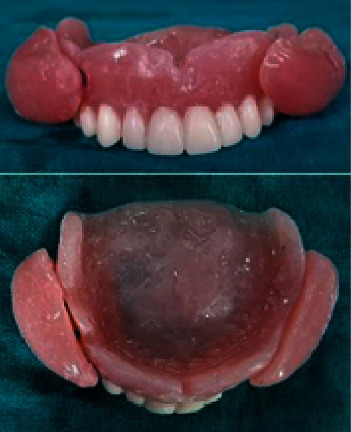
Maxillary denture with cheek plumper prosthesis (frontal view and intaglio surface).

**Figure 7:**
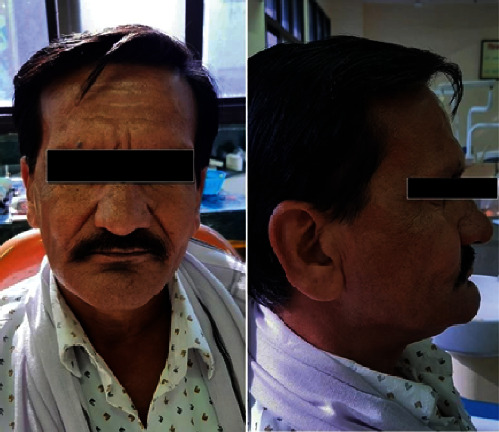
Straight profile and side profile of the patient with maxillary denture and cheek plumper prosthesis.

1. Primary impressions of maxillary and mandibular arches were made using modelling plastic impression compound (DPI pinnacle®), and custom trays were fabricated using autopolymerizing acrylic resin
2. Border moulding was done using green stick modelling plastic impression compound, and definitive impressions were made using zinc oxide eugenol impression paste
3. Jaw relations were recorded; teeth setting followed by try-in was done to check for occlusion, esthetics, and phonetics
4. Following try-in, cheek plumpers were made using green stick and impression compound in the ratio of 7 : 3 and were attached over the buccal flange of the waxed up maxillary denture in the premolar-molar region using magnetic attachments on either side for trial in the same appointment. The adapted plumpers were inspected extraorally for adequacy of cheek support and contour and interference with functional movements
5. Significant change in facial esthetics was seen after attaching cheek plumpers and was readily accepted by the patient
6. Magnets were removed from the cheek plumpers, and both the plumpers were processed using heat cure acrylic resin by reverse flasking (Figure 2)
7. After deflasking, cured denture and cheek plumpers were retrieved, trimmed, finished, and polished
8. Two 2 mm deep and 5 mm diameter holes on either side were made on the buccal flanges of the maxillary denture and corresponding area of cheek plumpers (Figures 3 and 4)
9. Magnets (Magfit™ DX600) (Table 1) were incorporated in the denture as well as cheek plumpers with autopolymerizing acrylic resin, and complete polymerization was ensured by placing it in a pressure pot. Magnet specifications are shown in Figure 5
10. After inserting the complete denture, plumpers were attached to the maxillary denture (Figure 6) and adequate clearance from the occlusal table was verified (Figure 6)
11. Attachment and removal of cheek plumper were demonstrated, and necessary instructions were given to the patient. He was asked to visit for regular follow-ups (Figure 7)

## 3. Discussion

The contour of the jaw bones, underlying teeth, and the soft tissues along with muscles around the teeth strongly influence the appearance of the lower half of the face [4]. Loss of teeth causes resorption of the alveolar ridge and loss of muscle tonicity. The apparent loss of subcutaneous fat, buccal pad of fat, and elasticity of connective tissue produces sunken cheeks. Rectification of drooping of cheeks can be done by different methods like reconstructive plastic surgery, injecting botulinum toxin (BOTOX) in the facial muscles, and various types of prosthesis [5]. Plastic surgery may be contraindicated in patients with a systemic disease. Moreover, it is a traumatic procedure and leaves behind a postsurgical scar.

Conventional cheek plumpers are single-unit prosthesis with extensions on either side of the posterior flange of denture base. Its continuous use leads to muscle fatigue and decreased retention of the prosthesis [6]. Muscle fatigue can be prevented if the patient has the option of removing cheek plumpers when experiencing discomfort. Therefore, in the present case, a detachable plumper prosthesis was planned to reduce weight of the prosthesis and facilitate easy placement and removal. Various attachments like magnets, press stud fasteners, orthodontic elastic modules, stud attachments, and wires can be used to attach cheek plumpers with the denture [7, 8]. Magnets facilitate automatic reseating because of the magnetic force and easy detachment of plumper prosthesis making it easy to clean. It can provide a constant amount of retentive force even after a number of insertion and removal cycles of the prosthesis. Its disadvantage is having poor corrosion resistance with oral fluids and so may require encapsulation with relatively inert alloy. Periodic patient recall is essential to evaluate the attachments and their replacement, when required.

## 4. Limitations

Magnets cannot be used in patients allergic to metal. The patients need to be apprised regarding the fact that the magnetic field used in MRI tests damages the magnetic assembly. The patients need to remove dentures for MRI tests. The magnetic assembly should be kept away from high temperatures of more than 150°C.

## 5. Conclusion

Cheek plumpers are simple to fabricate and provide a noninvasive and cost-effective treatment option to enhance facial esthetics in patients with sunken cheeks, thereby improving the patient's psychological well-being. Magnet-retained detachable cheek plumper is a modification of the conventional technique of supporting the slumped tissues due to ageing.

## Figures and Tables

**Table 1:** Magfit DX is a new thin-type, uncoated magnetic attachment with improved wear resistance. The ellipsoidal outer lip of the magnetic assembly assures firm fixation to the denture base.

		Fe	Nd	Cr	Ni	Others
Magnetic assembly	Magnet	65%	29%	—	—	6%
	Yoke/disc	79%	—	19%	—	2%
	Nonmagnetic part	68%	—	16%	12%	4%

Keeper unit	Keeper	79%	—	19%	—	2%
	Surface	68%	—	30%	—	2%
	Holder	68%	—	16%	12%	4%
